# The Association of the Sequence of Immunotherapy With the Survival of Unresectable Pancreatic Adenocarcinoma Patients: A Retrospective Analysis of the National Cancer Database

**DOI:** 10.3389/fonc.2020.01518

**Published:** 2020-09-02

**Authors:** Saber Amin, Michael J. Baine, Jane L. Meza, Chi Lin

**Affiliations:** ^1^Department of Radiation Oncology, University of Nebraska Medical Center, Omaha, NE, United States; ^2^Department of Biostatistics, College of Public Health, University of Nebraska Medical Center, Omaha, NE, United States

**Keywords:** pancreatic adenocarcinoma, immunotherapy, chemotherapy, radiation therapy, overall survival, national cancer database, treatment sequence

## Abstract

**Background:** Immunotherapy has shown great success in various malignancies. However, its efficacy in pancreatic ductal adenocarcinoma (PDAC) remains a challenge, and the lack of understanding about the appropriate timing of immunotherapy with other standard-of-care cancer treatments may be one of the causes. The objective of the current study is to investigate the impact of the timing of immunotherapy with chemotherapy and radiation therapy (RT) on the overall survival (OS) of PDAC patients who did not receive surgical resection of the pancreatic tumor.

**Materials and Methods:** Patients with pancreatic adenocarcinoma who did not receive surgical resection of the pancreatic tumor were identified from the National Cancer Database (NCDB). Cox proportional hazard models were employed to compare the OS between patients who received immunotherapy with chemotherapy or RT with a different sequence of treatment. The multivariable analysis was adjusted for age of diagnosis, race, sex, place of living, income, education, treatment facility type, insurance status, and year of diagnosis.

**Results:** In total, 705 patients received chemotherapy and immunotherapy, while 226 received radiation therapy and immunotherapy. In the multivariable analysis, there was no significant difference in the OS of patients who started immunotherapy 31–90 days before the start of chemotherapy with a hazard ratio (HR) of [HR:1.057 (CI: 0.716–1.56; *p* < 0.781)] and patients who started immunotherapy 91–180 days before the start of chemotherapy [HR: 0.900 (CI: 0.584–1.388; *p* < 0.635)] compared to patients who started chemotherapy and immunotherapy within 30 days of each other. There was also no significant difference in the OS of patients who started RT> 30 days before the start of immunotherapy [HR: 0.636 (CI: 0.346–1.171; *p* < 0.146)] and patients who started immunotherapy > 30 days before the start of RT [HR: 0.660 (CI: 0.328–1.329; *p* < 0.246)] compared to patients who started RT and immunotherapy within 30 days of each other.

**Conclusion:** The sequence of immunotherapy with chemotherapy or RT was not associated with improved OS. Future studies with a larger subgroup sample size investigating the impact of the timing of immunotherapy with chemotherapy and RT on the OS of PDAC patients who do not receive surgical resection of the pancreatic tumor are needed.

## Introduction

Pancreatic ductal adenocarcinoma (PDAC) represents 3.2% of all cancer cases, but it is responsible for 7.2% of all cancer deaths in the United States (1). It is predicted that PDAC will become the second leading cause of cancer deaths by 2030, after lung cancer (2). The median 5-year survival rate is 28–30% for localized diseases and only 8% for all stages. Due to the lack of sensitive biomarkers for early detections, more than 80% of the patients present with a locally advanced (non-resectable) disease (3, 4).

Surgery is the only curative treatment, but unfortunately, only 15–20% of patients present with cancer that is amenable to resection (5). Resectable patients undergo curative surgery followed by a combination of fractionated radiation therapy (RT) and chemotherapy as adjuvant therapies, while unresectable patients receive chemotherapy or chemoradiation (4). Surgery is associated with a median OS of 28 months when used with adjuvant gemcitabine plus capecitabine (6). Median survival time of up to 54 months has been reported with adjuvant modified FOLFIRINOX in resected pancreatic cancer patients (7, 8). Capecitabine-based chemoradiation therapy was associated with a median OS of 15.2 months in patients with locally advanced pancreatic cancer patients (8). The median OS of metastatic PC is 11 months in patients who receive FOLFIRINOX (9). Due to the minimal effectiveness of the available treatments, new effective therapies for PC are urgently needed.

In recent years, immunotherapy has shown great success in various malignancies, but is not approved by the FDA for the treatment of PDAC and is used in the clinic as a last attempt after the failure of the current standard treatments (10–16). Due to the negative results of the monotherapy with checkpoint inhibitors trials in PDAC, most recent trials have focused on combining immunotherapy with chemotherapy and RT (17–24). These trials reported a partial or objective response rate of 10.5–29%, and stable disease rate of 21–42% (20–22). One of the trials reported a median overall survival of 7.4 months (CI: 5.8–9.4) much longer compared to historical control (20). These findings indicate the activity and measurable outcomes associated with the use of immunotherapy in unresectable PDAC.

Chemotherapy and RT cause the release of neoantigens and upregulation of inflammatory cytokines, which are critical for the optimal function of immune cells stimulated by immunotherapy (25–29). Chemotherapy and RT increase tumor-specific T cell infiltration, decrease T regulatory cells, and suppress Myeloid-derived suppressor cells (MDSCs), and can have synergistic interaction with immunotherapy (30–32). Immunotherapy was associated with tumor regression and improved OS in preclinical studies of PDAC when used in combination with other treatments (33, 34). For the optimal effect of immunotherapy, the sequence of immunotherapy with chemotherapy and RT need to be balanced with the transient immunosuppressive impact of these treatments.

The available data seems to justify either the concurrent or delayed administration of immunotherapy (35). A retrospective study of non-small cell lung cancer (NSCLC) patients found a higher response rate for immunotherapy followed by chemotherapy compared to chemotherapy followed by immunotherapy (53.4 vs. 34.9%) (36). In another study, patients who received stereotactic radiasurgery (SRS) within 5.5 months after anti-cytotoxic T-lymphocyte-associated protein 4 (CTLA4) had improved control rate compared to ≥5.5 months (37). Contrarily a few other studies reported better results when SRS was administered either before immunotherapy or concurrently (38–41). The PACIFIC trial of NSCLC reported that the use of immunotherapy within 2 weeks of chemoradiation is associated with better OS compared to after 2 weeks (42). Patients with brain metastases (BMs) from melanoma who received whole-brain RT plus SRS before Ipilimumab had better median OS compared to Ipilimumab before SRS or concurrently with (26 vs. 6 vs. 18) months (38).

The majority of these studies were not designed to investigate the treatment sequence due to the absence of comparison group, had small sample size, only looked into SRS or RT in BMs, and only included Ipilimumab. Current ongoing clinical trials are designed to deliver immunotherapy concurrently with RT or after RT, ignoring the reports that giving anti-CTLA4 before palliative RT may improve response rate (43, 44).

Our own published data indicated that immunotherapy is associated with improved OS compared to no immunotherapy in unresectable PDAC patients (45). Immunotherapy was associated with improved OS compared to no immunotherapy [HR: 0.866 (CI: 0.800–0.937; *P* < 0.0001)], immunotherapy plus chemotherapy compared to chemotherapy alone [HR: 0.848 (CI: 0.766–0.938; *P* < 0.0001)], and chemoradiation plus immunotherapy compared to chemoradiation alone [HR: 0.813 (CI: 0.707–0.936; *P* < 0.0001)] (45). Therefore, the current study is a follow-up study of this published study. Determining the most appropriate sequence of immunotherapy with chemotherapy, RT, and chemoradiation may be an essential next step to maximize the effect of immunotherapy on the OS of PDAC patients.

There is no consensus about the sequence of immunotherapy with RT, chemotherapy, and chemoradiation, and there is no study that has investigated the sequence of immunotherapy with other cancer treatments in PDAC as most of the trials of immunotherapy in PDAC are in their early phases. The objective of this study is to investigate the impact of the sequence of immunotherapy with chemotherapy, and chemoradiation on the OS of PDAC patients using the National Cancer Database (NCDB) in an attempt to determine the appropriate treatment sequence that could be used to mitigate the immunosuppressive effects of the current treatments and maximize the impact of immunotherapy.

## Methods

### Data Source

The data for this study was obtained from the National Cancer Database (NCDB), which is a joint program of the Commission on Cancer of the American College of Surgeons and the American Cancer Society. The National Cancer Database captures 70% or more of newly diagnosed malignancies in the United States annually. The institutional review board evaluation was not obtained because the database provides de-identified data.

### Study Population

**Figure 1:**
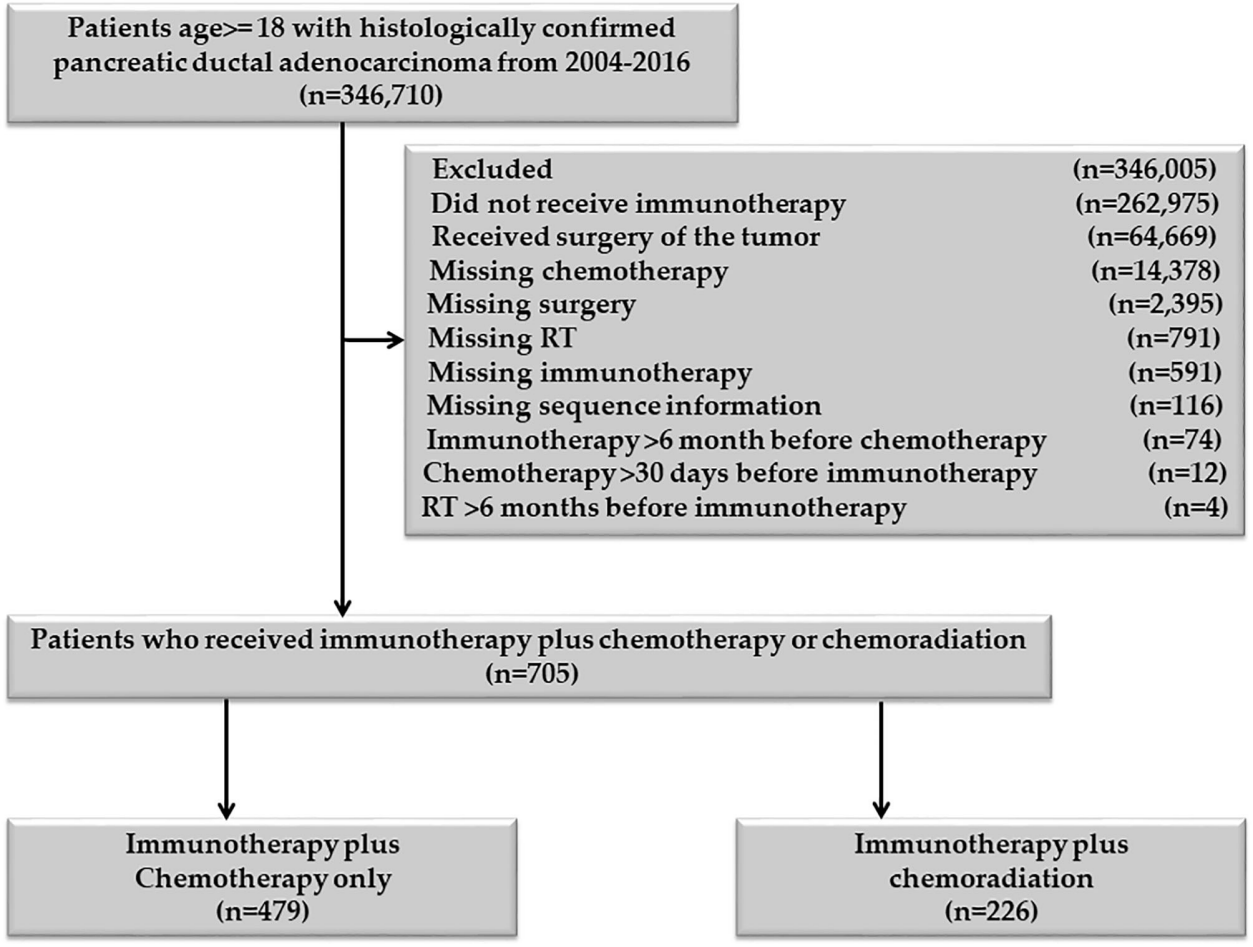
Patient selection flowchart of the study participants.

Patients age 18 or older, diagnosed with PDAC between 2004 and 2016, were included in the study. Patients who received definitive surgery of the primary pancreatic cancer and those who had missing information on RT, chemotherapy, and immunotherapy were excluded Figure 1. Patients with unknown or missing information about other covariates were not included in the adjusted multivariable analysis. The chemotherapy plus immunotherapy treatment sequence was divided into chemotherapy and immunotherapy within 30 days of each other, immunotherapy 31–90 days before chemotherapy, and immunotherapy 91–180 days before chemotherapy. There was not enough sample for chemotherapy >30 days before immunotherapy (*n* = 12), so this category was excluded. The RT and immunotherapy treatment sequences were divided into RT and immunotherapy within 30 days of each other, 30 < RT ≤ 180 days before immunotherapy, and 30 < immunotherapy ≤ 180 days before RT. Patients who started immunotherapy > 6 months before chemotherapy or chemotherapy >6 months before immunotherapy were excluded. Patients who started RT >6 months before immunotherapy or immunotherapy >6 months before RT were also excluded. All patients in the RT plus immunotherapy group also received chemotherapy, so this group represents the chemoradiation plus immunotherapy cohort.

### End Points

The primary outcome of the current study was overall survival (OS), which was calculated from the date of diagnosis to the date of death from any cause. Those alive or lost to follow up were censored at the date of the last contact. We also reported the treatment patterns related to the use of immunotherapy.

### Statistical Analyses

Descriptive statistics for categorical and continuous variables are reported. The association of various demographic and tumor-related factors with the type of treatment sequence was tested using the chi-square test of association. Kaplan–Meier curves were used to report the median OS, and log-rank tests were utilized to report the difference in the OS between the treatment sequences. Cox regression analysis was used to assess the association of treatment sequence with the OS of the patients. The multivariable analysis was adjusted for age at diagnosis, sex, race, income, education, insurance, place of living, hospital type, comorbidity score, and year of diagnosis. The estimated hazard ratios with associated 95% confidence intervals (CI) from the univariable and multivariable Cox analyses were reported. A *P* < 0.05 was considered for a significant level. The SAS 9.4 software was used for the analysis.

## Results

### Chemotherapy and Immunotherapy With or Without RT

In total, 705 patients were eligible for the final analysis of this group. Among them, 621/705 (88.09%) started chemotherapy and immunotherapy within 30 days of each other, 41/705 (5.82%) started immunotherapy 31–90 days before the start of chemotherapy, and 43/705 (6.10%) started immunotherapy 91–180 days before the starting chemotherapy. Among 621 patients who started chemotherapy and immunotherapy within 30 days of each other 470/621 (75.68%) started the two treatments on the same day, 525/621 (84.54%) started within 2 days, and 551/621 (88.57%) started within seven days of each other. The last two proportions are cumulative proportions. There were only 12/705 (1.7%) patients who started chemotherapy 31–90 days before immunotherapy. None of the patients started chemotherapy 91–180 days before immunotherapy. We excluded patients who started chemotherapy 31–90 days before immunotherapy (“*n* = 12”), as this small number was not enough to make a separate category.

The median age at diagnosis for the entire cohort was 64.00, with a range at 21–90 years. The median age at the diagnosis was 64.00 (21–90) years for patients who started chemotherapy and immunotherapy within 30 days of each other, 65.00 (44–83) years for the group who started immunotherapy 31–90 days before chemotherapy, and 64.00 (40–79) years for patients who started immunotherapy 91–180 days before chemotherapy. The majority of the patients were White, had high school degrees, had income ≥$35,000, were insured, living in the urban areas, were treated in academic hospitals, and had a comorbidity score of zero. There was no association between the baseline characteristics of the patients and the treatment sequence except the hospital type and the year of diagnosis. Among patients who started chemotherapy and immunotherapy within 30 days of each other, 63.93% were treated at academic facilities. In patients who started immunotherapy 31–90 days before the start chemotherapy, 46.34% were treated at academic hospitals, while 72.09% of the patients who started immunotherapy 91–180 days before the start of chemotherapy were treated at academic hospitals. The proportion of patients who were diagnosed in 2011 and later were 46.22, 63.41, and 83.72% for those who started chemotherapy and immunotherapy within 30 days of each other, started immunotherapy 31–90 days before chemotherapy, and those who started immunotherapy 91–180 days before the start chemotherapy. The baseline characteristics are provided in Table 1.

**Table 1:** Baseline characteristics of the sequence of immunotherapy with chemotherapy in PDAC patients with no surgery.

**Variable**		**CTx and immx within**	**Immx 31–90 days bf CTx**	**Immx 91–180 days bf CTx**	**Total**	***P***
		**30 days of each other**	***n* = 41 (5.82%)**	***n* = 43 (6.10%)**	***n* = 705**	
		***n* = 621 (88.09%)**				
Age at diagnosis (mean)		64.0 (21–90)	65.0 (44–83)	64.0 (40–79)	64.0 (21–90)	
Sex	Male	354 (57.00)	24 (58.54)	23 (53.49)	401 (56.88)	0.881
	Female	267 (43.00)	17 (41.46)	20 (46.51)	304 (43.12)	
Race	White	538 (87.48)	37 (90.24)	40 (95.24)	615 (88.11)	0.615
	Black	56 (9.11)	3 (7.32)	1 (2.38)	60 (8.60)	
	Other	21 (3.41)	2.44	1 (2.38	23 (3.30)	
	Unknown	6	0	1	7	
Education	≥13% NHD	216 (35.06)	11 (27.50)	10 (23.26)	237 (33.91)	0.194
	<13%	400 (64.94)	29 (72.50)	33 (76.74	462 (66.09)	
	Unknown	5	1	0	6	
Income	≥$35,000	402 (65.26)	28 (71.79)	29 (67.44)	459 (65.76)	0.686
	<35,000	214 (34.74)	11 (28.21)	14 (38.56)	239 (34.24)	
	Unknown	5	2	0	7	
Living place	Urban	587 (97.83)	38 (97.44)	42 (100.00)	667 (97.94)	0.616
	Rural	13 (2.17)	1 (2.56)	0 (0.00)	14 (2.06)	
	Unknown	21	2	1	24	
Hospital type	Academic	390 (63.93)	19 (46.34)	31 (72.09)	440 (63.40)	0.037
	Community	220 (36.07)	22 (53.66)	12 (27.91)	254 (36.60)	
	Unknown	11	0	0	11	
Insurance	Yes	572 (89.45)	40 (97.56)	39 (97.50)	651 (98.34)	0.832
	No	9 (10.55)	1 (2.44)	1 (2.50)	11 (1.66)	
	Unknown	41	0	2	43	
Charlson score	0	486 (78.26)	30 (73.17)	34 (79.07)	550 (78.01)	0.831
	1	108 (17.39)	8 (19.51)	8 (18.60)	124 (17.59)	
	≥2	27 (4.35)	3 (7.32)	1 (2.33)	31 (4.40)	
Year of diagnosis	2004–2010	334 (53.78)	15 (36.59)	7 (16.28)	356 (50.50)	0.0001
	2011–2016	287 (46.22)	26 (63.41)	36 (83.72)	349 (49.50)	

*CTx, chemotherapy; Immx, immunotherapy; bf, before; NHD, no high school degree*.

**Figure 2:**
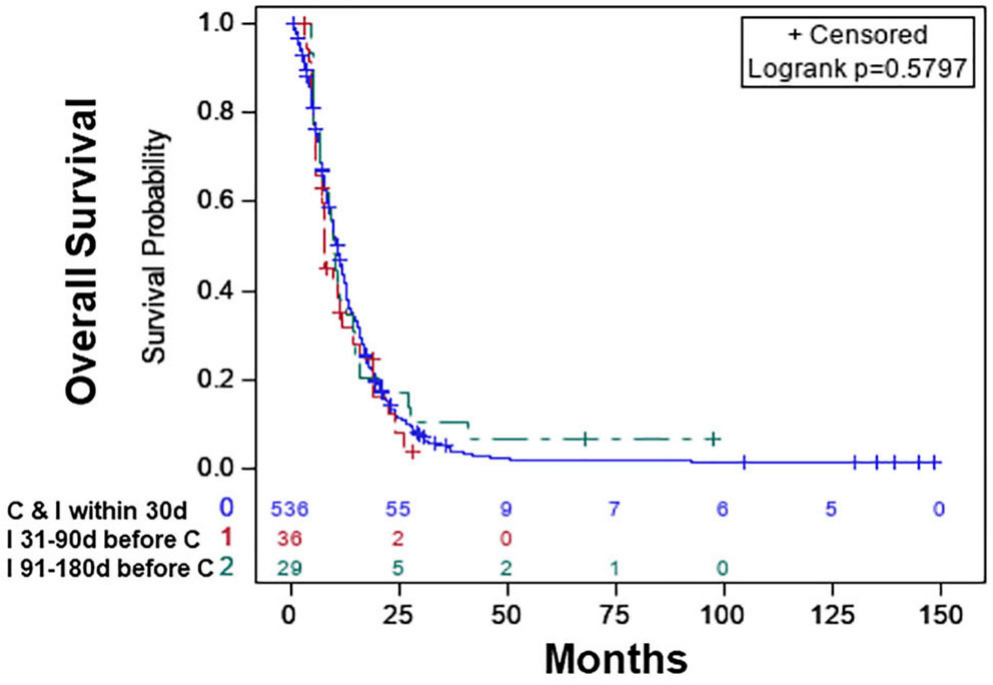
Overall survival of patients with chemotherapy plus immunotherapy with or without radiation therapy: chemotherapy and immunotherapy started within 30 days of each other (blue), immunotherapy started 31–90 days before the start of chemotherapy (red), immunotherapy started 91–180 days before the start of chemotherapy (green).

Based on the KM curves, the median OS of the treatment categories was not significantly different from each other (Figure 2). The median OS was 10.68 (CI: 9.79–11.66) months for patients who started chemotherapy and immunotherapy within 30 days of each other, 7.82 (CI: 5.85–11.93) months for patients who started immunotherapy 31–90 days before the start of chemotherapy, and 9.72 (6.67–14.62) months for patients who started immunotherapy 91–180 days before the start of chemotherapy Table 2.

**Table 2:** Median OS of chemotherapy and immunotherapy sequence groups.

**Variable**	**Median OS (95% CI)**
Chemotherapy and immunotherapy within 30 days of each other	10.68 (9.79–11.66)
Immunotherapy 31–90 days before chemotherapy	7.82 (5.85–11.93)
Immunotherapy 91–180 days before chemotherapy	9.72 (6.67–14.62)

In the multivariable Cox Proportional analysis (Table 3) adjusted for the age at diagnosis, sex, race, education, income, hospital type, comorbidity score, and year of diagnosis, there was no significant difference in the OS of patients who started immunotherapy 31–90 days before the start of chemotherapy [HR:1.057 (CI: 0.716–1.56; *p* < 0.781)] compared to patients who started chemotherapy and immunotherapy within 30 days of each other. There was also no difference in the OS of patients who started immunotherapy 91–180 days before the start of chemotherapy [HR: 0.900 (CI: 0.584–1.388; *p* < 0.635)] compared to patients who started chemotherapy and immunotherapy within 30 days of each other. The 1-year survival rates were 44% (CI: 40–48%) for patients who started chemotherapy and immunotherapy within 30 days of each other, 32% (CI: 16–48%) for those who started immunotherapy 31–90 days before starting chemotherapy, and 38% (CI: 20–56%) for patients who started immunotherapy 91–180 days before starting chemotherapy. The results did not change when the analysis was restricted to patients who only received chemotherapy plus immunotherapy (Table 3). The results also did not change when the analysis was restricted to only patients who received chemoradiation plus immunotherapy (Table 3). When we combined patients who started immunotherapy 31–90 days before chemotherapy with patients who started immunotherapy 91–180 days before chemotherapy (sequential use) and compared their OS with patients who started chemotherapy and immunotherapy within 30 days of each other (concomitant use), the results stayed the same. We also performed a propensity-matched analysis and compared the OS of the sequential and concomitant groups and did not find any difference in the OS of the two groups (data not shown).

**Table 3:** Univariable and multivariable cox regression analysis of the sequence of chemotherapy and immunotherapy.

		**Univariate analysis**		**Multivariable analysis**	
		**HR (95% CI)**	***P***	**HR (95% CI)**	***P***
**A: Chemotherapy plus immunotherapy regardless of RT (*****n*** **= 705)**					
CTx plus immunotherapy	CTx and Immx within 30 days	Ref		Ref	
	Immx 31–90 days before CTx	1.179 (0.815–1.705)	0.384	1.057 (0.716–1.561)	0.781
	Immx 91–180 days before CTx	0.901 (0.611–1.329)	0.598	0.900 (0.584–1.388)	0.635
**B: Only chemotherapy plus immunotherapy sample (*****n*** **= 479)**					
CTx plus immunotherapy	CTx and immx within 30 days	Ref		Ref	
	Immx 31–90 days before CTx	1.166 (0.762–1.784)	0.478	1.151 (0.731–1.812)	0.544
	Immx 91–180 days before CTX	0.984 (0.638–1.516)	0.941	0.952 (0.595–1.523)	0.838
**C: Chemoradiation plus immunotherapy sample (*****n*** **= 226)**					
CTx plus immunotherapy	CTx and immx within 30 days	Ref		Ref	
	Immx 31–90 days before CTx	0.969 (0.455–2.065)	0.936	0.860 (0.360–2.053)	0.734
	Immx 91–180 days before CTX	0.561 (0.228–1.377)	0.207	0.549 (0.165–1.826)	0.328

*CTx, chemotherapy; Immx, immunotherapy; RTx, radiation therapy; Ref, reference*.

### Radiation Therapy and Immunotherapy (Chemoradiation Plus Immunotherapy)

Among the 226 patients who received chemoradiation plus immunotherapy, 177/226 (78.32%) started RT and immunotherapy within 30 days of each other, 34/226 (15.04%) started RT > 30 days before starting immunotherapy, and 15/226 (6.64%) started immunotherapy > 30 days before starting RT. Importantly, among those who started RT and immunotherapy within 30 days of each other, 107/177 (60.45%) started the two treatment on the same day, 140/177 (79.66%) started the two treatments within 2 days from each other, and 153/177 (86.44%) patients started the two treatment within a week of each other indicating a pattern of care that clinicians are in favor of administrating the two treatment close to each other. Among the 34/226 (15.04%) patients who started RT >30 days before immunotherapy, 17/34 (50.0%) started RT 31–90 days before the start of immunotherapy, and 17/34 (50.0%) started RT 91–180 days before immunotherapy. Among the 15/226 (6.64%) patients who started immunotherapy >30 days before RT, 8/15 (53.33%) started immunotherapy 31–90 days before RT, and 7/15 (46.67%) started immunotherapy 91–180 days before RT. We did not make each of these a separate category due to the small sample size.

The median age of this cohort was 62.0 (33–85) years. The median age of those who started RT and immunotherapy within 30 days of each other was 61.0 (33–85), while median age of the patients who started RT >30 days before starting immunotherapy was 64.0 (80–37) years, and patients who received immunotherapy >30 days before the start of RT was 70.0 (47–80). Except for hospital type, comorbidity score, and year of diagnosis, no other variables were associated with the treatment sequence of RT and immunotherapy. Among patients who started RT and immunotherapy within 30 days of each other, 78.29%% were treated at academic hospitals, while 75.76% of the patients who started RT >30 days before immunotherapy and 33.33% of patients who started immunotherapy >30 days before RT were treated at academic hospitals. Among the patients who started RT and immunotherapy within 30 days of each other, 84.18% had comorbidity score of zero, while 73.53% of the patients who started RT >30 days before the start of immunotherapy, and 46.47% of the patients who started immunotherapy >30 days before the start of RT had comorbidity score of zero.

Among the patients who started RT and immunotherapy within 30 days of each other only 16.38% were diagnosed between 2011 and 2016, while 85.29% of the patients who began RT >30 days before the start of immunotherapy, and 60.00% of the patients who started immunotherapy >30 days before the start of RT were diagnosed between 2011 and 2016.

**Figure 3:**
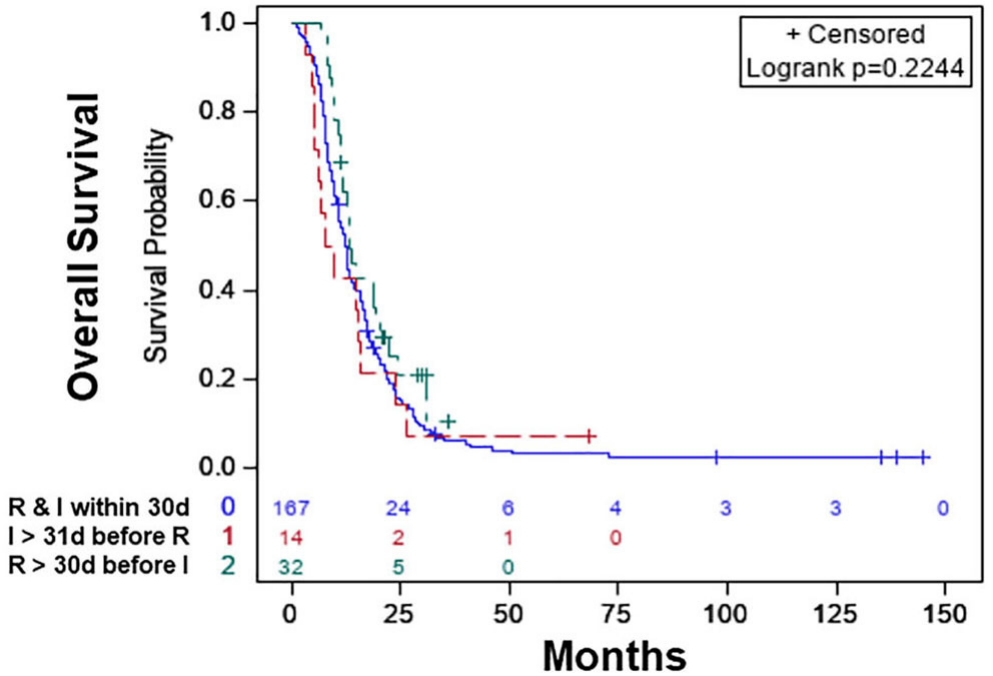
Overall survival of patients with radiation therapy plus immunotherapy with or without chemotherapy: radiation therapy and immunotherapy started within 30 days of each other (blue), immunotherapy started >30 days before the start of radiation therapy (red), radiation therapy started >30 days before the start of immunotherapy (green).

The characteristics of the patients are shown in Table 4. Based on KM, there was no significant difference in the median OS of the treatment sequence groups (Figure 3; *p* < 0.497). The median OS was 12.39 (CI: 10.84–13.54) months for patients who started RT and immunotherapy within 30 days of each other only, 13.27 (CI: 11.20–19.19) months patients who started RT >30 days before the start of immunotherapy, and 8.54 (CI: 5.09–15.67) months patients who started immunotherapy >30 days before the start of RT (Table 5).

**Table 4:** Baseline characteristics of the sequence of radiation therapy with immunotherapy in PDAC patients with no surgery.

**Variable**		**RT and immx within 30 days**	**Immx >30 days before RT**	**RT >30 days before immx**	**Total**	***P***
		**of each other**	***n* = 15 (6.64)**	***n* = 34 (15.04)**	***n* = 226**	
		***n* = 177 (78.32)**				
Age at diagnosis (mean)		61.0 (33–85)	70.0 (47–80)	64.0 (80–37)	62.0 (33–85)	
Sex	Male	99 (55.93)	10 (66.67)	15 (44.12)	124 (54.87)	0.285
	Female	78 (44.07	5 (33.33)	19 (55.88)	102 (45.13)	
Race	White	154 (89.53)	12 (80.00)	29 (85.29)	195 (88.24)	0.367
	Black	12 (6.98)	2 (13.33)	5 (14.71)	19 (8.60)	
	Other	6 (3.49)	1 (6.67)	0 (0.00)	7 (3.17)	
	Unknown	5	0	0	5	
Education	≥13% NHD	58 (32.95)	7 (46.67)	11 (32.35)	76 (33.78)	0.550
	<13%	118 (67.05)	8 (53.33)	23 (67.65)	149 (66.22)	
	Unknown	1	0	0	1	
Income	≥$35,000	123 (69.89)	8 (53.33)	24 (70.59)	155 (68.89)	0.402
	<35,000	53 (30.11)	7 (46.67)	10 (29.41)	70 (31.11)	
	Unknown	1	0	0	1	
Place of living	Urban	168 (97.67)	15 (100.00)	33 (100.00)	216 (98.18)	0.566
	Rural	4 (2.33)	0 (0.00)	0 (0.00)	4 (1.82)	
	Unknown	5	0	1	6	
Hospital type	Academic	137 (78.29)	5 (33.33)	25 (75.76)	167 (74.89)	0.0006
	Community	38 (21.71)	10 (66.67)	8 (24.24)	56 (25.11)	
	Unknown	2	0	1	3	
Insurance	Yes	148 (98.67)	14 (100.00)	33 (97.06)	195 (98.48)	0.701
	No	2 (1.33)	0 (0.00)	1 (2.94)	3 (1.52)	
	Unknown	27	1	0	28	
Charlson score	0	149 (84.18)	7 (46.67)	25 (73.53)	181 (80.09)	0.001
	1	23 (12.99)	5 (33.33)	8 (23.53)	36 (15.93)	
	≥2	5 (2.82)	3 (20.00)	1 (2.94)	9 (3.98)	
Year of diagnosis	2004–2010	148 (83.62)	6 (40.00)	5 (14.71)	159 (70.35)	0.0001
	2011–2016	29 (16.38)	9 (60.00)	29 (85.29)	67 (29.65)	

*RT, radiation therapy; Immx, immunotherapy; bf, before; NHD, no high school degree*.

**Table 5:** Median OS of RT and immunotherapy sequence groups.

**Variable**	**Median OS (95% CI)**
RT and immunotherapy within 30 days of each other	12.39 (10.84–13.54)
RT > 30 days before immunotherapy	13.27 (11.20–19.19)
Immunotherapy > 30 days before RT	8.54 (5.09–15.67)

In the multivariable analysis, there was no significant difference in the OS of patients who started RT >30 days before the start of immunotherapy [HR: 0.636 (CI: 0.346–1.171; *p* < 0.146)] compared to patients who started RT and immunotherapy within 30 days of each other. The OS was also not different between patients who started immunotherapy >30 days before the start of RT [HR: 0.660 (CI: 0.328–1.329; *p* < 0.246)] compared to patients who started RT and immunotherapy within 30 days of each other (Table 6). The 1-year survival rates were 51% (CI: 44–59%) for patients who started RT and immunotherapy within 30 days of each other, 43% (CI: 17–69%) for those who started immunotherapy >30 days before starting RT, and 62% (CI: 61–79%) for patients who started RT >30 days before starting RT. The results were the same when the within 30 days, 31–90 days, and 91–180 days classification was used. When we combined patients who started immunotherapy >30 days before RT with patients who started RT >30 days before RT (sequential use) and compared their OS with patients who started RT and immunotherapy within 30 days of each other (concomitant use), the results stayed the same. We also performed a propensity-matched analysis and compared the OS of the sequential and concomitant groups and did not find any difference in the OS of the two groups (data not shown).

**Table 6:** Univariate and multivariable cox analysis of the sequence of radiation therapy and immunotherapy.

**Variables**		**Univariate analysis**		**Multivariable analysis**	
		**HR (95% CI)**	***P***	**HR (95% CI)**	***P***
Radiation therapy plus immunotherapy	RT and immx within 30 days	Ref		Ref	
	RT >30 days bf Immx	0.713 (0.467–1.089)	0.117	0.636 (0.346–1.171)	0.146
	Immx >30 days bf RT	1.169 (0.663–2.060)	0.589	0.660 (0.328–1.329)	0.245

*Immx, immunotherapy; RT, radiation therapy; Ref, reference*.

## Discussion

To our knowledge, the current study is the first and the most extensive research on reporting treatment patterns in the use of immunotherapy and Comparing the impact of the timing of immunotherapy with chemotherapy and RT on the OS of PDAC patients who did not receive definitive surgery of the pancreatic cancer.

This study provides information about the timing pattern of immunotherapy treatment in PDAC patients. The findings indicate that the majority of patients receive immunotherapy within 30 days of chemotherapy or RT. The results also suggest that clinicians tend to start immunotherapy close to the start of chemotherapy or RT. As noticed, the majority of the patients started immunotherapy on the same day with starting chemotherapy or RT. Current clinical guidelines favor the concurrent use of immunotherapy with chemotherapy or RT; however, starting immunotherapy on the same day with chemotherapy or RT may not deliver the optimal benefits as chemotherapy, and RT both cause transient immunosuppression. Starting immunotherapy during that window of systemic and local immunosuppression may minimize the synergetic effect of the interaction of immunotherapy with chemotherapy and RT. The majority of the patients who received immunotherapy with 30 days of chemotherapy or RT were treated at academic centers, and these centers tend to recommend the concurrent use of immunotherapy with chemo and RT. Current ongoing clinical trials which some of these centers may be participating in are also administering the concomitant use of immunotherapy with other treatments. Data are lacking to either confirm or oppose the current treatment sequence used in these clinical trials.

In the current study, the treatment sequence of immunotherapy with chemotherapy and RT was not associated with improved OS. Our own published data indicated that immunotherapy is associated with improved OS when combined with chemotherapy or chemoradiation. Based on the findings of that data, we decided to investigate the timing of immunotherapy with other cancer treatments and see if the timing of immunotherapy matters. However, the results of the current study indicate that the improved OS associated with the use of immunotherapy in combination with chemotherapy or chemoradiation therapy does not depend on the sequence of the treatments.

The optimal time of immunotherapy may depend on the mechanism of the immunotherapy drug and the cancer type (35). For example, a preclinical study of colorectal carcinoma found that the optimal timing for the anti-CTLA4 blockade is before RT, while for anti-OX40 agonists, the best time is after RT (46). These findings have been supported by clinical studies and case series of metastatic melanoma, gastrointestinal cancers, NSCLC, lymphoma, and head and neck cancer patients in which patients received immunotherapy first and then received RT or chemotherapy (36, 47–52). On the other hand, a few other studies which only included brain metastasis (BMs) patients from melanoma reported better results when SRS was administered either before immunotherapy or concurrently (38–41).

Our findings are consistent with the results of other studies in which there was no difference in the OS of BMs patients when RT was delivered before immunotherapy or after immunotherapy or if RT was administered concurrently with immunotherapy or sequentially (53–55).

The negative results of the study may be in part due to the small sample size of some of the treatment sequence groups, especially for immunotherapy plus RT cohort. The insignificant results of the sequence of RT with immunotherapy may be due to the use of a low dose of conventional RT fraction in most of these patients. The majority of the patients received conventional RT with 1.8–2 Gray per fraction, and past reports have suggested that higher fractional doses such as those provided with SBRT are required to improve immunotherapy when combined with RT. It is also possible that the benefit of immunotherapy with RT is drowned by using immunotherapy with chemo and vice versa. However, when we restricted the analysis to patients who only received chemotherapy and immunotherapy, the results did not change. It is also possible that the sequence of immunotherapy with other treatments such as chemotherapy and RT does not matter, and immunotherapy is associated with improved OS, as found in published data (45).

Immunotherapy is not a standard-of-care treatment in pancreatic cancer outside of clinical trials. However, some patients are receiving Immunotherapy. It is possible that patients who received Immunotherapy were taking part in a clinical trial. In the current study, 254/705 (36.0%) were treated at community hospitals, an indication that some patients are receiving immunotherapy without taking part in a clinical trial. It is also possible that immunotherapy was recommended in patients who have exhausted many lines of standard-of-care treatments.

The strength of this study is the relatively large sample size, which allows for adjusting for various critical patient and tumor-related factors. However, the study is not without several limitations. The limitations include selection bias, miscoding, lack of information on the cause of death, lack of information about the type of immunotherapy, and if a single or combined immunotherapy was administered, and lack of detailed information on the use of multi-agent chemotherapy. Another limitation is the exclusion of patients who received chemotherapy >30 days before immunotherapy. Only (“*n* = 12”) patients were in this group who were excluded, and a separate group with only 12 patients was not enough to be included in the analysis. The small sample size for some treatment sequence groups in both immunotherapy plus chemotherapy and immunotherapy plus RT was another limitation of the study.

## Conclusions

To our knowledge, the current study is the first and the most extensive research that has compared the association between the timing of immunotherapy with chemotherapy and RT and the OS of PDAC patients. There was no association between the treatment sequence of immunotherapy with chemotherapy or RT and the OS of the patients. Future studies with a large sample size for each subgroup of the treatment sequences are needed to investigate the association of the timing of immunotherapy with chemotherapy and RT with the OS of PDAC patients.

## Data Availability Statement

The raw data supporting the conclusion of this article will be available by the corresponding author upon reasonable request.

## Ethics Statement

Ethical review and approval was not required for the study on human participants in accordance with the local legislation and institutional requirements. Written informed consent for participation was not required for this study in accordance with the national legislation and the institutional requirements.

## Author Contributions

SA: design, analysis, manuscript writing, review, and editing. MB: design, review, and editing. JM: analysis, review, and editing. CL: design, writing, review, and editing. All authors read and approved the final manuscript.

## Conflict of Interest

The authors declare that the research was conducted in the absence of any commercial or financial relationships that could be construed as a potential conflict of interest.
